# The Impact of a Randomized Community-Based Intervention on the Awareness of Women Residing in Lebanon Toward Breast Cancer, Cervical Cancer, and Intimate Hygiene

**DOI:** 10.3390/healthcare12232422

**Published:** 2024-12-03

**Authors:** Rim Chehab, Rimla Abboud, Mariane Bou Zeidan, Chelsy Eid, Giovanni Gerges, Cecile Z. Attieh, Said Btadini, Dana O. Kazma, Sophia M. Bou Chahine El Chalouhi, Mohammad Abi Haidar, Maram M. Abdulaal, Ralph Maatouk, Karen Maatouk, Sarah El Khoury, Malek N. Nassar, Béchara El Asmar, Mirna N. Chahine

**Affiliations:** 1 Faculty of Medical Sciences, Lebanese University, Hadath P.O. Box 3, Lebanon; r.chehab.2@st.ul.edu.lb (R.C.); rimla.abboud.2@st.ul.edu.lb (R.A.); m.bouzeidan.1@st.ul.edu.lb (M.B.Z.); chelsy.eid@st.ul.edu.lb (C.E.); giovannigerges123@gmail.com (G.G.); ralphmaatoukmd@gmail.com (R.M.); karenmaatouk@gmail.com (K.M.); 2 Faculty of Medicine & Medical Sciences, University of Balamand, Koura Campus, Tripoli P.O. Box 100, Lebanon; cecile.attieh@std.balamand.edu.lb; 3 Department of Biology, Faculty of Arts & Sciences, University of Balamand, Dekouaneh Campus, Sin-el-Fil P.O. Box 55251, Lebanon; said.btadini@std.balamand.edu.lb (S.B.); dkazma@std.balamand.edu.lb (D.O.K.); sophia.elchalouhi@std.balamand.edu.lb (S.M.B.C.E.C.); mohammad.abihaidarb@std.balamand.edu.lb (M.A.H.); maram.abdulaal@std.balamand.edu.lb (M.M.A.); 4 Lebanese Association of the Knights of Malta (Order of Malta Lebanon), Vanlian Bldg, 6th Fl. City Rama Str. Dekwaneh, Beirut P.O. Box 11-4286, Lebanon; elkhourysarah@yahoo.com (S.E.K.); drmaleknassar@yahoo.fr (M.N.N.); bechara.elasmar@hdf.usj.edu.lb (B.E.A.); 5 Department of Obstetrics and Reproduction, Hotel-Dieu de France Hospital, Achrafieh, Beirut P.O. Box 11-5190, Lebanon; 6 Faculty of Medicine, Saint Joseph University, Beirut P.O. Box 17-5208, Lebanon; 7 Department of Cardiology, Hotel-Dieu de France Hospital, Achrafieh, Beirut P.O. Box 11-5190, Lebanon; 8 Foundation-Medical Research Institutes (F-MRI®), Achrafieh, Beirut P.O. Box 64, Lebanon; 9 Foundation-Medical Research Institutes (F-MRI®), 1211 Geneva, Switzerland

**Keywords:** women’s health, breast cancer, cervical cancer, intimate hygiene, intervention, awareness campaign, knowledge, preventive measures

## Abstract

Background/Objectives: Women’s health represents an integral component of public health. With breast cancer being the first worldwide and cervical cancer the fourth most common cancer among women, and while habits of intimate hygiene remain mediocre, it is crucial to address these issues. We aimed to evaluate the efficacy of a community-based intervention on the knowledge and preventive measures (K&P) of women toward breast cancer, cervical cancer, and intimate hygiene. Methods: This longitudinal multicentric prospective interventional study was conducted on women residing in Lebanon aged 18–83 years old. The awareness sessions took place either in person or online to address these three topics, covering the eight governorates of Lebanon. Our sample included women both from the general population and from Order of Malta Lebanon (OML)’s centers and mobile medical units. A stratified randomized sampling method was implemented using age and governorates. Women were interviewed before (pre-test) and after (post-test) the awareness session. The K&P score and the improvement post-intervention were represented in a function of all the study variables. A *p* ˂ 0.05 was found statistically significant. Results: A total of 657 women, with the majority being nonsmokers and having a bachelor’s degree as their highest level of education, completed surveys before and after the intervention, showcasing a significant overall K&P mean score improvement of 50.48% in the pre- vs. post-test (an average score of 22.01 ± 5.95 over 38 (57.93% of correct answers) vs. 33.12 ± 3.41 over 38 (89.58% of correct answers), respectively, *p* < 0.001). A significant difference was also noted between pre- and post-test (*p* < 0.001) in each of the three topics individually, with improvements of 52.39% for breast cancer, 60.00% for cervical cancer, and 22.27% for intimate hygiene. Conclusions: National awareness campaigns are key to shedding light on breast and cervical cancer matters and improving women’s reproductive health and intimate hygiene in Lebanon. Addressing the knowledge gaps and promoting early screening and healthy habits through national public health policies can empower women to protect their health and well-being.

## 1. Introduction

Important issues of women’s health, such as breast cancer, cervical cancer, and intimate hygiene, call for appropriate awareness and education. Breast cancer is the primary cause of cancer-related mortality and morbidity in women, according to epidemiological profiles that are consistent across practically all nations [1]. In 2020, breast cancer was at the top of the list of the most frequently diagnosed cancers globally, and it is expected to affect more than 3 million new people and cause 1 million deaths yearly by 2040 [2]. On the other hand, cervical cancer falls as the fourth most frequently diagnosed cancer in women, as well as the fourth most fatal cancer in women, with 311,365 deaths in 2018 [3]. These figures are particularly concerning in countries in the process of social transformation, where resources, access to care, and health literacy are all limited. Lack of information and adequate knowledge about breast [1,4,5] and cervical cancer [6,7,8,9,10], proper intimate hygiene habits [11,12], and related issues may lead to delayed diagnosis, limited treatment options, and increased health risks. Numerous factors, such as a lack of funding for research in these areas and the social class that restricts treatment alternatives might be blamed for these issues [7,11,13].

Breast cancer, cervical cancer, Human papillomavirus (HPV), and intimate hygiene, from bacterial vaginosis to douching and vaginal yeast infections, all fall under the title of “women’s health” [14]. And here is what is known about these topics and their implications in our region:

A 2019 study on breast cancer’s global incidence, mortality, and burden found that countries with low socioeconomic status had greater rates of mortality from breast cancer, despite having a lower incidence than countries with high socioeconomic status. It is worth noting that the Eastern Mediterranean Region topped that ranking. High mortality rates can be attributed to limited resources and a lack of national health strategies [15]. In the specific context of Lebanon, national awareness campaigns on breast cancer have been conducted annually since 2002 through various platforms, with emphasis on the importance of screening [16]. As for cervical cancer, awareness campaigns are less common; however, a study showed that only 35% of the 2255 Lebanese women that were included had performed at least one Pap smear [17]. As for intimate hygiene, a study has revealed that unhealthy habits are not uncommon in Lebanon [12]. Therefore, it was necessary to assess the knowledge of Lebanese women about these important health topics, especially that all three topics constitute a pivotal aspect of every woman’s health journey.

A study published on the breast self-exam (BSE) prevalence rate among Lebanese women in February 2023 [4] found that women in Lebanon need to be more aware of breast cancer to prevent late representations and reduce the associated mortality and morbidity. This study [4] and others [1,5] suggested that particular emphasis should be placed on the importance of expanding education and awareness programs. A study performed on 1312 Lebanese University students showed that their knowledge of cervical cancer, Papanicolaou (Pap) smear, and HPV was low; thus, more effort must be put into educational programs to raise awareness and lower the mortality rate from cervical cancer [6]. Furthermore, intimate hygiene routines are essential for a woman’s general health and well-being. It is crucial to understand proper hygiene practices, including sexually transmitted infections management, urinary tract infections prevention, and general intimate care. According to a study conducted in Lebanon by the Lebanese University, 25% of the women who filled out the survey said they had vaginal infections or discomfort due to bad intimate hygiene habits [12].

By educating women and emphasizing adherence to the recommendations for clinical breast examination, Pap test for cervical cancer screening, and general reproductive health check, awareness workshops serve as a platform for health promotion behavior [6]. Having highlighted the importance of tackling these issues, it is crucial to increase knowledge on these topics and on prevention methods among women residing in Lebanon. Thus far, there has been no study in Lebanon approaching breast cancer, cervical cancer, and intimate hygiene through a community-based intervention carried out by providing awareness sessions to women all over Lebanon and from different age categories.

With that being said, this research highlights the importance of monitoring women’s assimilation of awareness sessions and healthy habits for long-term impact. Covering all Lebanese territories, this research is vital to understanding how women interact and show interest in learning more about their health.

Our objective was to assess the impact of a community-based intervention on the knowledge and preventive measures (K&P) of women residing in Lebanon toward breast cancer, cervical cancer, and intimate hygiene by determining the overall levels of K&P toward these topics among women in Lebanon; evaluating the correlation between K&P and sociodemographic characteristics (age, area of residence, level of education, type of work, and others); identifying potential misconceptions regarding these health concerns; and assessing the efficacy of awareness sessions, both online and in person. Hence, we aimed to shed light on the importance of awareness campaigns focusing on women’s health and policy reinforcement.

## 2. Materials and Methods

**Figure 1:**
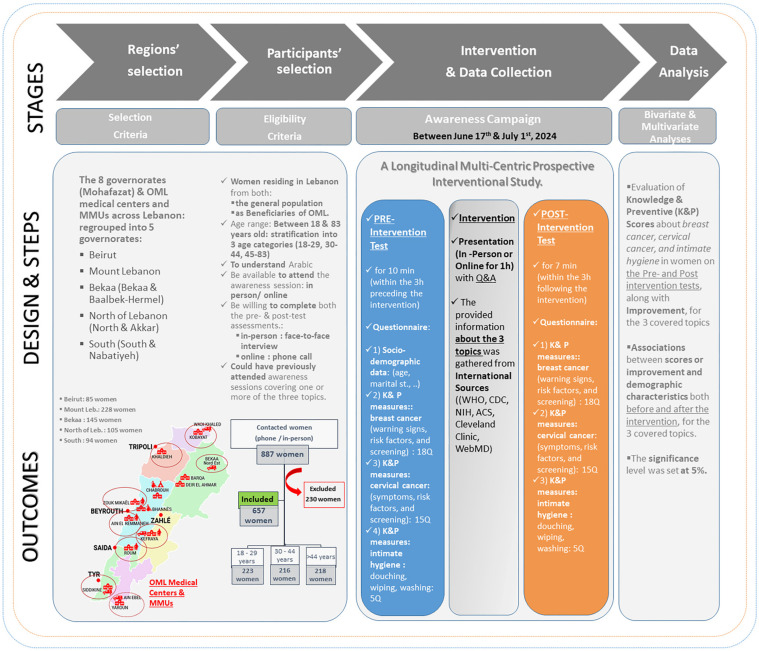
Study flow including the contents, methods, and times of the intervention. OML: Order of Malta; MMUs: mobile medical units; WHO: World Health Organization, CDC: Centers for Disease Control and Prevention, NIH: National Institutes of Health, ACS: American Cancer Society.

The methods followed the CONSORT reporting guidelines [18]. The study flow is represented in Figure 1.

### 2.1. Study Design and Population

#### 2.1.1. Study Design

Our study was a longitudinal multicentric prospective interventional study. This assessment used a pre-test and post-test approach. The pre-test and post-test, conducted either in person (face-to-face interview) or via phone call, occurred within a 3 h window before and after the awareness session, respectively. This study was part of a women’s health campaign organized by the Order of Malta (OML) for women from the general population and those beneficiaries of the different medical centers of OML across Lebanon. Face-to-face interviews or online sessions took place between 17 June and 1 July 2024, inclusively. Women involved in this study were stratified according to governorates and 3 age categories (18–29 y, 30–44 y, 45–83 y) in order to ensure representation.

In this design, a pre-test (before the intervention) was carried out through a survey (face-to-face interviews or over the phone) to assess the baseline of awareness (knowledge and preventive measures) of our population on breast cancer, cervical cancer, and intimate hygiene. Following that, the intervention consisted of a one-hour awareness session covering our 3 topics. This took place either on site (if pre- and post-tests were conducted in person) or online (if pre- and post-tests were conducted via phone call). Lastly, a post-test (after the intervention) was carried out using the same survey (face-to-face interviews or over the phone) to detect differences and changes occurring as a result of the intervention compared with the baseline obtained in the pre-test.

#### 2.1.2. Inclusion Criteria

The study included women residing in Lebanon from both the general population and the Order of Malta Lebanon (OML). The participants’ age ranged from 18 to 83 years old. Participants had to understand Arabic, be available to attend the awareness session, and be willing to complete both the pre-test and post-test assessments. Additionally, eligible candidates could have previously attended awareness sessions covering one or more of the three specified topics.

#### 2.1.3. Exclusion Criteria

The study excluded women residing outside Lebanon. Additionally, individuals younger than 18 or older than 83 years were not included. Women without internet access were ineligible for online sessions. Other criteria for exclusion encompassed unavailability to attend the awareness session and unwillingness to complete either the pre-test or post-test assessments.

#### 2.1.4. Experimental Intervention

In this study, participants from all age groups and all governorates were interviewed by trained medical students/midwives to fill out the pre-test in a window of 3 h before the intervention. The awareness session consisted of a unified oral PowerPoint presentation prepared and reviewed by doctors and/or midwives. The PowerPoint files were adopted and edited according to the Order of Malta template (OML communication department). Three PowerPoint presentations were prepared for each of the three topics, and each presentation took 20 min. The awareness session started with the breast cancer presentation, followed by the cervical cancer one, and lastly, the intimate hygiene presentation. Between each topic, 5–10 min of time were allocated for questions and answers. The participants were expected to attend all three of the sessions; if for any reason someone was not able to be there for all three of the topics, the post-test was not carried out with these participants (and results of the pre-test were not included). Both online and in-person sessions were equally interactive by involving participation of the attendees through a Q&A segment. Presentations were delivered in the same dynamics and level of engagement.

##### Information Used During the Awareness Session Presentation

The information provided during the awareness presentations was gathered from international sources (such as the World Health Organization (WHO), Centers for Disease Control and Prevention (CDC), National Institutes of Health (NIH), American Cancer Society, Cleveland Clinic, WebMD) [19,20,21,22,23,24,25,26,27,28,29,30], adapted to the population by the researcher, and reviewed by experts in the field (gynecologists, midwives). The doctors and the midwives presented the PowerPoint multiple times in various locations across different governorates in Lebanon (whether for OML beneficiaries or the general population) or virtually over Zoom platforms for women from the general population and beneficiaries of OML. The same PowerPoint presentations were given, both on site and online, covering all the important aspects of the three topics.

##### Questionnaire Used as Pre- and Post-Test

A structured questionnaire based on validated questionnaires [7,11,17,31,32] and information from our presentations [19,20,21,22,23,24,25,26,27,28,29,30] was used in this study. In the process of questionnaire validation, in addition to the Cronbach’s alpha (α value > 0.7) that assesses internal consistency, as explained in Section 2.3, a pilot study was conducted on 30 women who were not part of the sample to validate the understanding and clarity of the questionnaire items. At the end of this step, the questionnaire was modified as necessary [33]. The questionnaire, uploaded on a Google Form, was available in both the Arabic and English languages. By using the inverted method of Fortin, it was translated from English to Arabic [33]. After the submission of answers through the Google Form, the data were converted and stored in a confidential Excel sheet. A pre-test was conducted to validate the understanding and clarity of the questionnaire items with ten individuals who were not part of the sample. Then, the questionnaire was adjusted as necessary according to the feedback from the pre-test [34].

The questionnaire encompassed several sections:

- Section one gathered sociodemographic data (such as age, nationality, marital status, dwelling region, educational level, profession, medical history, weight, height, smoking status, attendance of any previous awareness campaign).
- Section two examined knowledge and preventive measures related to breast cancer, covering warning signs (such as lumps, bleeding, discharge, and redness), risk factors, and screening. This section comprised 5 main branches with a total of 18 questions.
- Section three addressed knowledge and preventive measures regarding cervical cancer, exploring symptoms, risk factors, and screening, divided into 7 main branches with a total of 15 questions.
- Section four focused on knowledge and preventive measures for maintaining proper intimate hygiene, including questions about douching, wiping, and washing, with a total of 5 questions.

##### 2.1.5. Sample Size

Based on the Slovin Formula, *n* = *N*(1 + *Ne*2), where N represents the Lebanese population, which was 5,489,739 in 2023, following the Index Mundi registry of the Lebanese population, with women representing around 50% of the population (N = 2,744,869), e represents the *p*-value of 0.05. Therefore, a minimum of 399 women had to fill out the questionnaire to be representative of the population in Lebanon.

The general population was targeted in all 8 governorates (Mohafazat) in Lebanon: Akkar, North, Beirut, Mount Lebanon, Bekaa, Baalbek-Hermel, Nabatiyeh, and South. However, since the population is unequally distributed, we regrouped them into 5 governorates: Beirut, Mount Lebanon, Bekaa (Bekaa and Baalbek-Hermel), North of Lebanon (North and Akkar), and South of Lebanon (South and Nabatiyeh).

As for beneficiaries of the Order of Malta (OML), women were contacted by phone according to a list provided by the OML covering all women registered with OML centers and mobile medical units (MMUs) distributed in the entire Lebanese territory (through 8 medical centers: Ain-el-Remmaneh (Beirut), Barqa (Bekaa Hermel), Khaldieh (North), Kobayat (North), Roum (South), Siddikin (South), Yaroun (South), and Zouk (Mount Lebanon) and 5 MMUs: Akkar (North), Kefraya (Bekaa Hermel), Khaldieh (North), Ras Baalbek (Bekaa Hermel), and Roum/Jezzin (South)), thus making this study a multicentric interventional study.

Whether recruited from the general population or from the OML beneficiaries’ list, women were randomly chosen, but their inclusion was made according to age and governorate in order to guarantee a representative sample of the population. This means that during the recruitment, the population was chosen after stratification into 3 age categories (18–29, 30–44, 45–83) within the 5 Lebanese governorates according to the distribution of the population in term of women’s age and the percentage of the population per governorate on the Lebanese territory, respectively.

#### 2.1.6. Sampling and Randomization

The randomization method used in our study followed a stratified random sampling. The population was stratified based on governorate (stratum), and then a random ID was assigned to each participant using the Excel formula for randomization (RAND). After organizing the data by governorate, samples were selected from each age category (18–29 y, 30–44 y, 45–83 y) to ensure representation from the stratum, improving precision, reducing sampling variability, and thus ensuring adequate representation.

### 2.2. Data Collection

#### 2.2.1. Procedures of Data Collection

- Step 1—Pre-test: Conducted by the interviewer, the pre-test consisted of a 10 min questionnaire collected over Google Forms within the 3 h preceding the awareness session. The questionnaire was filled via a face-to-face interview for the in-person sessions and over a phone call for the online sessions. It started with a section about sociodemographic characteristics, and then was aimed at testing the participants’ knowledge of our study’s three topics, without relying on the internet or any other source for the answers.
- Step 2—Intervention: The intervention consisted of awareness sessions that took place in person or online. The duration of the session was 1 h, divided into 20 min for each of the three topics. The floor was open for questions, both at the end of each topic and at the end of the whole presentation. The investigators (medical doctors) explained different points of the questionnaire, focusing on domains that were particularly missed in the pre-test questionnaire. An active engagement with attendees was ensured, regardless of it being conducted virtually or in an OML center/amphitheater setting.
- Step 3—Post-test: The post-test consisted of the same questionnaire as the pre-test (except without the sociodemographic characteristics section). This 7 min questionnaire was conducted by the same interviewer as the pre-test and was also collected over Google Forms. It was filled via a face-to-face interview or a phone call. It was aimed at testing the participants’ knowledge after having received all the relevant information in the awareness session (Figure 1).

#### 2.2.2. Measurements

Knowledge and preventive measures scores for breast cancer, cervical cancer, and intimate hygiene were computed based on correct and incorrect answers to 38 questions (18 items for breast cancer, 15 for cervical cancer, and 5 for intimate hygiene). Each correct answer received 1 point, and the overall knowledge and preventive measures score was derived from the sum of these points, with the median serving as the cutoff value for each section. Incorrect answers received 0 points.

For knowledge and preventive measures assessment, the computed scores were graded into categories and subcategories of “Limited” (Poor, Fair) to “Adequate” (Good, Excellent) levels of knowledge and preventive measures, according to the median of the scores and a modified form of the widely adopted Bloom’s cutoff points, with 80–100% (good K&P), 60–79% (moderate K&P), and less than 60% (poor K&P) [35,36,37,38,39], as shown in Table 1. These cutoff values were also based on previously published K&P studies from our laboratory [37,38,39] and elsewhere [35,36].

### 2.3. Statistical Analysis

The overall analysis of the data was performed using the SPSS 22 program. For normally distributed data, the mean and standard deviation were calculated; for data not normally distributed, median and range were calculated. Categorical variables were expressed as counts and percentages, and their proportions were compared using the chi-square test. As for the numerical variables, they were compared using ANOVA or *t*-test. A significance level of 0.05 was considered statistically significant. Knowledge and preventive measures scores pre- and post-intervention and continuous variables were presented with mean, standard deviation, minimum, and maximum values, while categorical variables were represented by frequency and percentage. The knowledge and preventive measures score was categorized to show poor, fair, good, and excellent knowledge and preventive measures about breast cancer, cervical cancer, and intimate hygiene in women and was represented as frequencies and proportions. Internal consistency (Cronbach’s alpha) (α) was assessed for all the scores, which were valid, with a Cronbach’s alpha value > 0.7 for K&P overall score (Cronbach’s alpha: 0.842), K&P breast cancer score (Cronbach’s alpha: 0.811), and K&P cervical cancer score (Cronbach’s alpha: 0.733). (Only the Cronbach’s alpha value for K&P intimate hygiene score was < 0.7). The improvement in knowledge and preventive measures was tested between the pre-knowledge and preventive measures score and the post-knowledge and preventive measures score using a Paired Samples *t*-test. Bivariate analysis, including Student’s *t*-test and ANOVA tests, was conducted to examine associations between scores and demographic characteristics both before and after the intervention. A multivariate analysis assessed factors influencing each score. The model included all the factors that were statistically associated with the dependent in the bivariate settings with a *p* value less than 0.1 (10%). The multivariate analysis was performed in two steps: the intermodal, that included all the variables, and the forward model, to eliminate the confounding factors. The significance level was set at 5%.

### 2.4. Ethical Information

Ethical approval was obtained from the Ethics Committee of the University Hospital Center Lebanese Geitaoui Hospital (Number: 2024-IRB-017; Date: 22 May 2024). Participants were informed that our study is voluntary and anonymous, and that all the gathered information was solely used for the study and remained confidential.

This study was conducted in accordance with Good Clinical Practice ICH Section 3 and the principles laid down by the 18th World Medical Assembly (Helsinki, 1964) [40] and all applicable amendments. Responses were confidential and were used only for research purposes. If participants agreed to participate voluntarily in our study, they were asked by the investigators to sign electronically an informed consent in Arabic, where a detailed explanation of the background, objectives, risks, and advantages of the study was provided.

## 3. Results

### 3.1. Population Characteristics

#### 3.1.1. General Characteristics of Participants in Our Study

Out of 887 women contacted through phone or in person, a total of 657 participants completed the survey, thus yielding an acceptance rate of 74%, in accordance with the eligibility criteria outlined above. The mean age of the sample was M = 38.05, SD = 14.07 years, with a minimum of 18 and maximum of 83 years old, with approximately an equal distribution among the age ranges. Most participants were between 18 and 29 years old (33.9%), followed by those over 44 years old (33.2%) and lastly, those between 30 and 44 years old (32.9%). Participants in this study were predominantly Lebanese women (95.1%), mostly married (56.6%), had a bachelor’s degree as their highest level of education (62.3%), and worked mostly either outside of the healthcare system (40.5%) or engaged in household labor (24.2%). The demographic details of our participants are presented in Table 2.

#### 3.1.2. Clinical Characteristics of Participants in Our Study

Out of the 657 participants, the majority (63.5%) were nonsmokers. The prevalent reported health conditions included hypertension, high cholesterol, obesity, and thyroid diseases (12.3%, 12.3%, 9.4%, and 9.1%, respectively). Notably, most of the participants (88.9%) had never previously attended awareness campaigns on breast cancer, cervical cancer, or intimate hygiene.

### 3.2. General Knowledge and Preventive Measures (K&P) Scores for Women’s Health: Difference Between Overall Pre-Test and Post-Test Scores and Their Improvement

**Figure 2:**
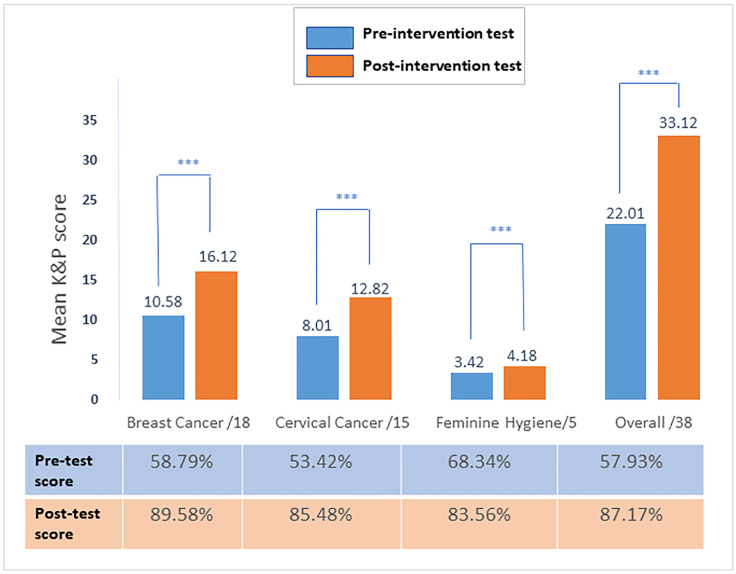
Overall, breast cancer, cervical cancer, and intimate hygiene K&P scores represented as mean and %. *** *p* < 0.001: statistically significant.

The mean overall score about these three topics was poor in the pre-test, being 22.01 ± 5.95 over 38 (57.93% of correct answers) vs. good in the post-test, being 33.12 ± 3.41 over 38 (87.17% of correct answers) (Figure 2, Supplementary Table S1), thus showing an improvement of 50.48%. A significant difference was noted between the pre-test and post-test (*p* < 0.001).

**Figure 3:**
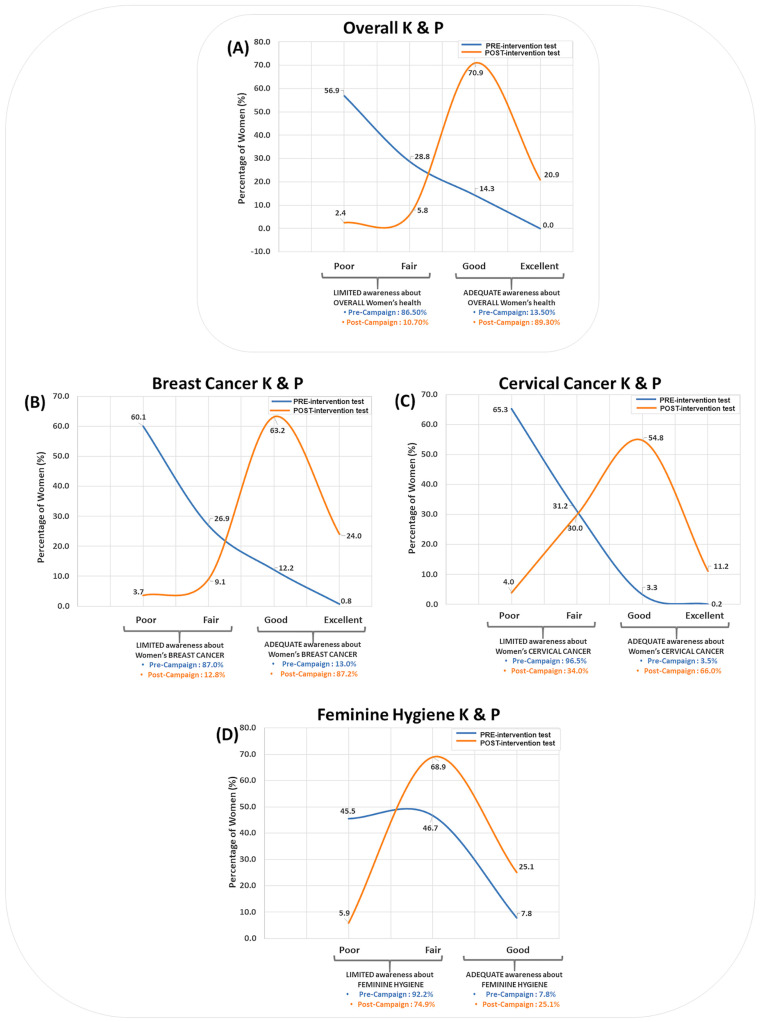
Graphs showing the mean percentage (%) of women’s answers before and after the awareness campaign about (**A**) the three topics overall, (**B**) breast cancer, (**C**) cervical cancer, and (**D**) intimate (feminine) hygiene. Percentages are represented according to categories (Limited/Adequate) and subcategories (Poor/Fair/Good/Excellent).

By considering the overall pre-test questions about the three topics, 85.7% of the participants had a “Limited” overall K&P (regrouped as poor overall K&P scores: 56.9% and fair overall K&P scores: 28.8%) while 14.3% of participants had an “Adequate” K&P. During the overall post-test, 8.2% of participants remained with a “Limited” K&P while 91.8% of participants received an “Adequate” overall K&P (regrouped as good overall K&P scores: 70.9% and excellent overall K&P scores: 20.9%), thus showing the beneficial effect of the intervention on the participants (Figure 3A, Supplementary Figure S1).

A schematic representation of the percentage (%) of women’s answers before and after the awareness campaign in each of the three topics, organized under subcategories “Poor/Fair/Good/Excellent”, is visualized in Supplementary Figure S1.

### 3.3. Differences in Parameters Between Pre-Test and Post-Test Regarding the Knowledge and Preventive Measures (K&P) About Breast Cancer

A total of 657 responses were recorded. The mean score of K&P about breast cancer was poor in the pre-test, being 10.58 ± 3.43 over 18 (58.79% of correct answers), vs. good in the post-test, being 16.12 ± 2.23 over 18 (89.58% of correct answers) (Figure 2, Supplementary Table S2), thus showing an improvement of 52.39% between the pre- and post-tests. A significant difference was noted between the pre-test and post-test (*p* < 0.001).

During the pre-test about breast cancer, 87.1% of participants had a “Limited” K&P (regrouped as poor K&P scores: 60.1% and fair K&P scores: 26.9%), while 12.9% of participants had an “Adequate” K&P. During the post-test about breast cancer, 12.8% of participants remained with a “Limited” K&P, while 87.2% of participants had an “Adequate” K&P (regrouped as good K&P scores: 63.2% and excellent K&P scores: 24.0%), thereby demonstrating the positive impact of this intervention (Figure 3B, Supplementary Figure S1).

**Figure 4:**
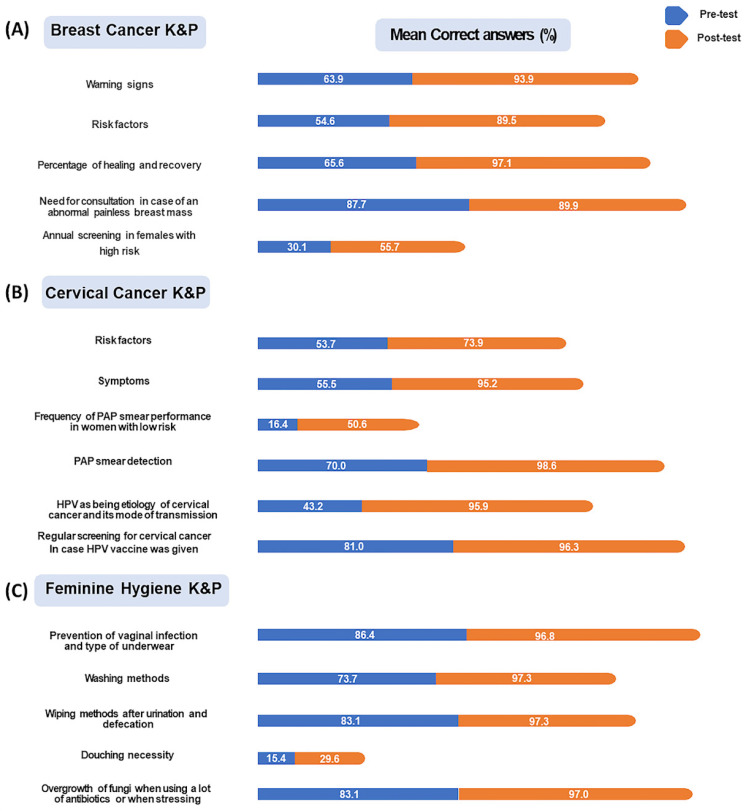
Mean correct answers of participants regarding questions about (**A**) breast cancer, (**B**) cervical cancer, and (**C**) intimate (feminine) hygiene recorded during pre- and post-intervention tests. Numbers are represented as percentage (%).

The difference was particularly emphasized in questions tackling warning signs; for example, 90.6% of people correctly identified a rash on or around the nipple as a warning sign in the post-test compared with only 51.4% in the pre-test (with an improvement of 76.04%), as well as 84.2% correctly identified early menstruation as a risk factor for breast cancer in the post-test, whereas only 16.1% of participants were able to do so in the pre-test (a 421.70% improvement) (Figure 4A). Further results are presented in Supplementary Table S2.

As for the factors that influenced the pre-test score of breast cancer K&P, the bivariate analysis revealed a significant disparity between having a higher score in the breast cancer test and marital status (*p* = 0.013), level of education (*p* < 0.001), working status (*p* = 0.005), and smoking status (*p* = 0.041). Indeed, significantly higher scores were recorded in unmarried (60.9% of correct answers) vs. married participants (57.1%). Participants with an MD/PhD scored the highest (73.9%). Additionally, people who work had a higher mean (60.91%) vs. those who do not work (56.78%), and participants with a healthcare background scored higher than participants with no healthcare background (71.47% vs. 58.85%) (*p* < 0.001). Furthermore, those who are nonsmokers or ex-smokers had a significantly higher pre-test score of breast cancer K&P than smokers (59.70% and 59.30% vs. 54.49%, respectively) (Table 3).

Similarly to the pre-test, the post-test score of K&P about breast cancer was significantly higher in unmarried participants (*p* = 0.007) (91.46% of correct answers) vs. married ones (88.14%), those with a higher education level (*p* < 0.001) (93.89%) vs. others, those who work vs. those who do not work (91.42% vs. 87.86%, respectively (*p* < 0.001)), participants with a healthcare background (91.67%) vs. others (*p* < 0.001), as well as nonsmokers or ex-smokers vs. smokers (89.99% and 92.06% vs. 84.76%, respectively (*p* < 0.001)). Thus, following the awareness session, the improvement in K&P about breast cancer was 50.19% in unmarried participants vs. 54.18% in married ones; 27.07% in those with an MD/PhD; 50.07% and 54.72% for participants who work vs. those who do not; 34.18% vs. 28.25% for those who have a healthcare background vs. those who do not; and 50.75% and 55.24% in non- and ex-smokers, respectively, vs. 55.53% in smoker participants. Moreover, post-test results were correlated with governorate residency (*p* = 0.016) being in Bekaa/Baalbek-Hermel, 16.61 ± 0.99 over 18 (92.30%); followed by Beirut, 16.35 ± 2.38 over 18 (90.85%); not suffering from obesity 16.26 ± 2.11 over 18 (90.32%) vs. obese participants (*p* < 0.001); and those without comorbidities, 16.37 ± 1.93 over 18 (90.93%) vs. those with co-morbidities (*p* < 0.001) (Table 3). This highlights the relationship between various factors and the K&P scores about breast cancer, emphasizing the need to focus on our population and effectively reach women with low K&P.

### 3.4. General Knowledge and Preventive Measures (K&P) Scores (Identification of Risk Factors and Symptoms) About Cervical Cancer: Difference Between Pre-Test and Post-Test Scores

The mean score of K&P about cervical cancer was poor in the pre-test, being 8.01 ± 2.93 over 15 (53.42% of correct answers) vs. good in the post-test, being 12.82 ± 1.52 over 15 (85.48% of correct answers) (Figure 2, Supplementary Table S3), thus showing an improvement of 60.00% between the pre- and post-test. A significant difference was noted between the pre-test and post-test (*p* < 0.001).

During the pre-test about cervical cancer, 96.5% of participants had a “Limited” K&P (regrouped as poor K&P scores: 65.3% and fair K&P scores: 31.2%), while 3.5% of participants had an “Adequate” K&P. During the post-test about cervical cancer, 33.9% of participants remained with a “Limited” K&P, while 66.1% of participants received an “Adequate” K&P (regrouped as good K&P scores: 54.8% and excellent K&P scores: 11.3%) (Figure 3C, Supplementary Figure S1). This consequently highlights the beneficial outcomes of this intervention. For instance, when participants were asked to identify if tingling is a symptom of cervical cancer, they showed an improvement of 313.10% (pre-test correct answers: 22.1% vs. post-test correct answers: 91.2%), whereas when they were asked “If I get HPV vaccine, I also need regular screening for cervical cancer”, an 18.98% improvement was noted (pre-test correct answers: 81.0% vs. post-test correct answers: 96.3%) (Figure 4B). More differences are shown in Supplementary Table S3.

As for the factors that influenced the pre-test score of cervical cancer K&P, the bivariate analysis showed that a statistical significance was observed in terms of nationality, with Lebanese women scoring lower than others (44.21% vs. 50.52%) (*p* = 0.032); educational level, with those having an MD/PhD scoring the highest (70.40%) vs. others (*p* < 0.001); and healthcare background, with those with a healthcare background scoring higher than those without (53.63% vs. 45.32%) (*p* < 0.001) (Table 4).

After the awareness session, this significant correlation appeared to be limited to governorate of residency (*p* = 0.009), with those living in the Bekaa/Baalbek-Hermel governorate scoring the highest (72.49%) (*p* < 0.001); educational level, with the highest level of education being an MD/PhD vs. never going to school (75.37% vs. 65.66%, respectively, *p* = 0.001); working status, with those working scoring significantly higher than those who do not (*p* = 0.008); and to smoking status, with non- and ex-smoker participants having higher scores than smokers (*p* = 0.008) (Table 4). Thus, following the awareness session, an improvement in K&P about cervical cancer was noted: 57.67% improvement for Bekaa/Baalbek-Hermel resident women and 24.26% and 80.56% improvement for participants with an MD/PhD vs. those who never went to school, respectively. In addition, those who do not smoke and are ex-smokers improved by 59.76% and 62.90%, respectively, compared with 57.07% in smoker participants. This underscores the association between different factors and the K&P scores related to cervical cancer, stressing the importance of focusing on our population and successfully engaging women with low K&P.

### 3.5. General Knowledge and Preventive (K&P) Measures Scores for Screening and Healthy Behavior of Intimate Hygiene: Difference Between Pre-Test and Post-Test Scores

The mean score of K&P about intimate hygiene was fair in the pre-test, being 3.42 ± 0.94 over 5 (68.3% of correct answers), vs. good in the post-test, being 4.18 ± 0.57 over 5 (83.6% of correct answers) (Figure 2, Supplementary Table S4), thus showing an improvement of 22.27%. A significant difference was noted between the pre-test and post-test (*p* < 0.001).

Out of the 657 participants, 45.5% of them had poor scores about intimate hygiene before the intervention, while 5.9% of them recorded poor scores after the intervention. Inversely, fair K&P scores about intimate hygiene were recorded by 46.7% and 68.9% of the participants before and after the intervention, respectively. In the same trend, good K&P scores about intimate hygiene were recorded by 7.8% and 25.1% of the participants before and after the intervention, respectively (Figure 3D, Supplementary Figure S1). This therefore emphasizes the beneficial effects of this intervention. For instance, participants were able to identify the right answers in most questions; for example, when asked “What do you use when washing?”, participants picked the following option, “the hand with water and a specific hygiene product”, more frequently in the post-test (97.3%) than in the pre-test (73.3%), thus showing an improvement of 32.02% (Figure 4C). More results are shown in Supplementary Table S4.

As for the factors that influenced the pre-test score of intimate hygiene K&P, the bivariate analysis showed that only the level of education was significantly correlated with the participants’ score in the intimate hygiene section before attending the session (*p* = 0.010). Those having an MD/PhD (76.6% of correct answers) scored significantly higher about intimate hygiene than those who never went to school (69.09%). That factor was no longer significant in the post-test, and three new factors that became significant in the post-tests were the smoking status (*p* = 0.032) and the presence or absence of obesity (*p* = 0.046) or co-morbidities (*p* = 0031). Indeed, those with the highest post-test scores of intimate hygiene K&P were nonsmokers (84.3%) followed by smokers and then ex-smokers (4.15 ± 0.66 over 5 (83.0%) and 4.07 ± 0.39 over 5 (81.4%), respectively). Also, significantly higher scores were observed between non-obese vs. obese (83.9% and 81.4%, respectively) and those without vs. with co-morbidities (84.3% and 82.3%, respectively) (Table 5). This underscores the association between multiple factors and the K&P scores associated with feminine hygiene, underscoring the necessity to focus on our target population and effectively engage women exhibiting low K&P.

### 3.6. General Knowledge and Preventive Measures (K&P) Scores for Women’s Health and the Difference Between Overall Pre-Test and Post-Test Scores and Their Improvement: Bivariate and Multivariate Analysis

As for the factors that influenced the overall pre-test score K&P, the bivariate analysis showed that a higher overall score was significantly associated with the level of education (having an MD/PhD scored higher than having never been to school (73.77% of correct answers vs. 51.44%, respectively (*p* < 0.001)); working status (working participants scored significantly higher than non-working ones (60.13% vs. 55.86%, respectively (*p* < 0.001)); participants with a healthcare background had a higher score than those who do not (68.78% vs. 58.44%, respectively, *p* < 0.001) (Table 6). When multivariate analyses were enrolled, the overall pre-test K&P about the three topics revealed that scores were better in working participants (B= 1.058; *p* = 0.024) and those with higher education levels (B = 1.547; *p* < 0.001) (Supplementary Table S5).

After attending the session (post-test), a similar trend was noted, with a higher overall score significantly associated with the level of education (MD/PhD, 34.77 ± 2.40 over 38 (91.49% of correct answers) vs. no school, 31.00 ± 6.19 over 38 (81.58%), *p* < 0.001); working type (*p* < 0.001); working status (working, 33.65 ± 3.18 over 38 (88.56%) vs. not working, 32.63 ± 3.53 over 38 (85.86%), *p* < 0.001); and having a healthcare background (33.63 ± 2.90 over 38 (88.51%) vs. others, *p* < 0.001). In addition, a higher overall score was found significantly associated with the following additional factors: Marital status, where single or unmarried participants scored higher than married ones (33.62 ± 3.21 over 38 (88.47%) vs. 32.75 ± 3.50 over 38 (86.18%), *p* = 0.001); residency governorate (those living in Bekaa/Baalbek-Hermel Governorate scored higher (33.83 ± 1.71 over 38 (89.02%)) than others (*p* < 0.001)); being a nonsmoker or ex-smoker (33.98 ± 3.32 over 38 (89.42%)) vs. being a nonsmoker (*p* < 0.001); being non-obese (33.32 ± 3.24 over 38 (87.69%)) vs. being obese (*p* < 0.001); having no co-morbidities (33.49 ± 2.97 over 38 (88.12%)) vs. having co-morbidities (*p* = 0.001); and not having attended any awareness session about breast cancer, cervical cancer, and intimate hygiene in the last 6 months (33.23 ± 3.83 over 38 (87.44%)) vs. others (*p* = 0.028) (Table 6). Multivariate analyses of the overall post-test K&P about the three topics showed that participants with higher scores were unmarried (B = −0.521; *p* = 0.047); workers (B = 0.571; *p* = 0.031); had a higher education level (B = 0.818; *p* < 0.001); were less obese (B = −0.840; *p* = 0.026); and had not attended any awareness session about breast cancer, cervical cancer, and intimate hygiene in the last 6 months (B= −0.920; *p* = 0.025) (Supplementary Table S5). These findings highlight the association between various factors and the overall K&P scores, emphasizing the significance of focusing on our population and successfully engaging women with low K&P.

## 4. Discussion

Breast cancer and cervical cancer have consistently ranked as the foremost causes of mortality among women, while intimate hygiene encompasses a wide spectrum of critical concerns, ranging from urinary tract infections (UTIs) to sexually transmitted infections (STIs). In our pursuit of promoting early diagnosis and treatment through awareness-raising, we conducted a multicentric interventional study. Participants underwent a pre-test, engaged in informative sessions covering the three pivotal topics, and concluded with a post-test to evaluate the intervention’s effectiveness. Our findings indicate a significant increase, with scores improving by 50.48% from the subcategory “Poor” (57.93% of correct answers) to “Good” (87.17% of correct answers) or from the category “Limited” to “Adequate”, between the overall pre- and post-tests about the three topics together. Specifically, the percentage of participants’ correct answers increased from the pre- to post-tests K&P about breast cancer, from 58.79% to 89.58%, respectively; cervical cancer, from 53.42% to 85.48%, respectively; and intimate hygiene, from 68.34% to 83.56%, respectively.

Similar studies to ours around the globe have reported limited knowledge about breast cancer, cervical cancer, or intimate hygiene:

### 4.1. Breast Cancer

The study conducted on Lebanese women in Beirut in 2018 tackling knowledge, attitude, and practices regarding breast cancer found that participants scored highly on knowledge of breast cancer symptoms (72.8 ± 24.7%) and poorly concerning knowledge about curability (49.6 ± 25.7%) [5]. This could implicate the need to focus on treatment and prognosis of breast cancer when addressing the general population, since knowing how to detect breast cancer from its symptoms is not enough; it needs to be complemented by action-taking, and being aware of the high chance of curability, if treated, could encourage women to follow up more on their annual mammograms. In a low-income area of Colombia, a study reported poor knowledge at baseline about breast cancer risk factors, such as family history of breast cancer (9.7% vs. 82.3% in our study), nulliparity (3.2% vs. 22.1% in our study), and late-onset menopause (6.5%) [41]. This could be justified by the expected lower level of education in low-income areas.

In the Promoting Early Presentation (PEP) intervention, 21.9% of the intervention group correctly identified older age as a risk factor for breast cancer three years post-intervention, compared with 7.7% of the usual care group (*p* ≤ 0.001) [42]. However, in our study, despite the intervention, responses about young age as a risk factor remained unchanged at around 78%. This may be due to the lack of attention, with participants falsely assuming they were being asked about old age, which is the correct answer. Actually, a study on young women in Malaysia showed the intervention group sustained good knowledge of breast cancer and breast self-exam (BSE) six months and twelve months following the intervention [43]; this could support the effectiveness of interventions in maintaining a good level of knowledge in the long term. Furthermore, concerning assessing the impact of online awareness sessions, a study conducted by Tuna et al. showed a significant increase in knowledge 1 to 6 months after an online educational session on BSE [44]. This could serve as a tool to support online awareness sessions as an alternative to in-person sessions when needed, as the former seem to be just as efficient, if not more.

As for the factors associated with the knowledge score about breast cancer, the Malaysian study found that young women still had limited knowledge about breast cancer due to a perceived lower likelihood of having it [43]. This is opposed to our findings, where age was not significantly associated with better knowledge about breast cancer following the awareness session. This could imply that all participants were equally interested and attentive and scored better than before the awareness session. Another factor that has been shown to be associated with the knowledge score is the education level of the population, as reported in a Bangladesh study [45], a factor similarly identified in our study (*p* < 0.001 for both pre- and post-tests), where education level affected participants’ responses, specifically in participants with higher levels of education (MD, PhD), as scores increased from 13.30 over 18 (73.88%) in the pre-test to 16.90 over 18 (93.89%) in the post-test, with a 27.07% improvement in correct answers, once again showcasing the effect of the education level. Likewise, women with a working status demonstrated an enhanced level of knowledge attributable to their advanced educational qualifications as recorded in their pre-test results, and this was reinforced post-intervention by their exposure to diverse professional experiences and continuous learning opportunities available through their employment. As for the unmarried women who had a higher knowledge score than married ones, this difference can be attributed to the additional time and focus that unmarried women can dedicate to learning; thus, they may possess a greater capacity to comprehend these topics, as demonstrated in the post-test results.

### 4.2. Cervical Cancer

The Lebanese study performed on female university students on cervical cancer, HPV, and the Pap smear showed they had generally low knowledge concerning these topics, including the use of the Pap smear and the frequency of doing this test in addition to HPV, its transmission, and its relationship with cervical cancer, among other factors as well [6]. This is particularly alarming, as HPV is a sexually transmitted infection, a topic university students should be aware of, with its manifestations and outcomes. Less than 25% of nurses in Nigeria recognized HPV vaccination as a preventive measure for cervical cancer at baseline (pre-test) as reported by Ndikom et al. [46], whereas in our study, 81.0% correctly identified in the pre-test that a Pap smear is still needed even with positive HPV vaccination status. This is a positive finding that could mirror the awareness of the HPV vaccine in our community. A study on Hispanic patients demonstrated increased knowledge of the Pap smear and HPV [47], comparably to our study, where an increase in the correct answers on the Pap smear and all questions on HPV was noted, having more than 96% correct answers in the post-test. This poses an interesting aspect of whether the increase in the knowledge will increase the number of HPV vaccinations and Pap smears. In rural Nepal, participants who received the educational sessions on cervical cancer showed a high turnout for free cervical cancer screening [48], a component that, if assessed, could have added value to our research, evaluating how more women will show up for screening when performed for free, thus removing any financial constraint.

As for factors associated with K&P about cervical cancer, Ford et al. found that demographic variables did not affect overall knowledge scores [49]. In contrast, our study identified that educational level of the participants, for instance, significantly affected the level of knowledge in both the pre-test and post-test (*p* < 0.001 and *p* = 0.001, respectively), reflecting that the educational process might expose residents of Lebanon to more knowledge about this topic. Papa et al. linked their participants’ understanding of the Pap test to their high education and presumed that awareness sessions could be equally or even more beneficial to less-educated women [50], thus showing the accessibility of the material presented. This can be also stressed through our study, as the improvement in scores of participants with the highest levels of education was 24.46%, but for participants that did not go to school, the improvement was much higher (80.56%), suggesting that participants with lower educational levels have more room for improvement. Furthermore, the working status was shown to be a factor associated with the knowledge pre-test score. However, this factor was no longer affecting the answers significantly in the post-test, which emphasizes the impact of the awareness session to approximate the level of knowledge about the discussed topics between different working statuses.

### 4.3. Intimate Hygiene

In Lebanon, a cross-sectional observational study performed with female patients and nurses revealed a significant lack of awareness concerning hygiene practices, with many of them taking regular intimate baths, douching, and using wet wipes or intimate deodorant sprays [11]. This could be due to false advertising of intimate hygiene products alongside the historical shame that comes with female genital health. The Zanzibar-led research had equal findings to our study, as it showed a significant increase in the K&P score in the post- vs. pre-test for intimate hygiene [51].

In the study of Charafeddine et al. on preconception health conducted on 7290 high school children in Lebanon, being female or in a private school was a significant predictor of higher scores in both the pre-test and post-test (*p* < 0.001) [52]. Similarly, in our study, having the highest level of education predicted having better scores in the pre-test (*p* = 0.006) about intimate hygiene, in addition to other factors. For instance, the type of work seemed to affect the scores more significantly in the post-test (*p* = 0.049) [52]; people working in healthcare seemed to score lower than those who do not. In the study about preconception health among young adolescents in Lebanon, participants in younger grades showed more improvement in knowledge and scored higher in the post-test, whereas participants in higher grades had higher knowledge only at baseline [52], showing that young participants could have more to learn and improve their knowledge.

The study tackling sexual and reproductive health communication suggested that older age (50–59 years) and higher education were associated with better communication with their adolescents [51]. However, in our study, which focused on intimate hygiene—closely related to reproductive health—only higher education had a significant impact on the pre-test (*p* = 0.006), whereas the post-test was significant only for being nonsmokers, less obese, and with no comorbidities. With the level of education no longer affecting the answers significantly in the post-test, we can see the benefits of the awareness session to approximate the level of knowledge about the discussed topics between different educational classes.

### 4.4. Overall K&P Scores and Predictors

**Figure 5:**
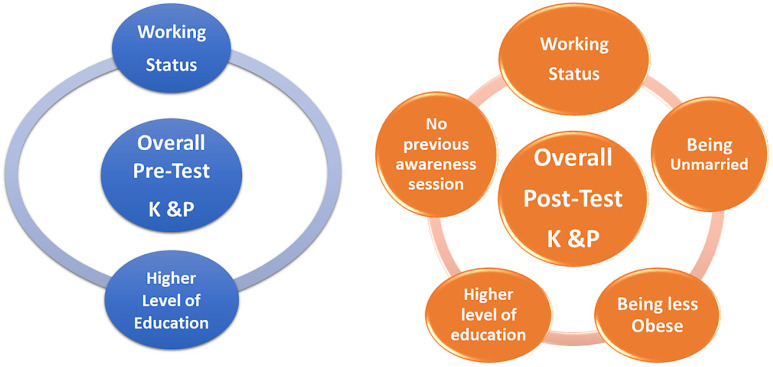
Predictors significantly associated with overall pre-test and post-test K&P scores about breast cancer, cervical cancer, and intimate hygiene.

Predictors significantly associated with overall (the three topics together) K&P levels before (pre-test) and after the campaign (post-test) are summarized in Figure 5.

The pre-test total scores showed that the highest degree of education, type of work, working status, and having a background in healthcare were important factors in bivariate analysis. Only having a working status and having the greatest level of education were significant enough to be included in the multivariate analysis. This seems likely, especially given that the material in the awareness session concerns every woman, regardless of her working status or type of work, as it affects her overall health and well-being. Furthermore, the multivariate analysis confirmed employees to be more knowledgeable due to their higher educational attainment.

In the post-test overall scores, the significant factors in bivariate analysis were the highest level of education; type of work; working status; having a healthcare background; marital status (being unmarried); smoking status (non- or ex-smoker); in addition to being not smoking, not obese, and not having previously attended an awareness session on any of our topics. Out of these characteristics, the greatest level of education had enough weight to remain relevant in the multivariate analysis, along with working status and being unmarried, non-obese, and not having attended previous sessions. Participants who had not previously attended awareness sessions were paying more attention and appeared to be interested in expanding their knowledge. Furthermore, regarding the observed difference between married and unmarried participants, this could be attributed to the fact that, in general, unmarried women would enjoy a level of freedom and flexibility, with no commitments or responsibilities associated with marriage, which provides a more relaxed environment for exploration and embracement of new knowledge during the awareness sessions. Finally, in terms of overall health and risk factors for breast and cervical cancer, obesity and smoking are both important issues to consider. This was confirmed in our multivariate analysis, which revealed that healthy BMI and nonsmoker status without co-morbidities had a greater impact on improvement in post-test outcomes.

### 4.5. Weaknesses and Strengths

Despite the impacts of the study, it was not without its *limitations*. The survey was subject to social desirability bias, especially since it tackled topics perceived as sensitive by our communities. The survey approach had limitations in reaching illiterate or underprivileged individuals without phone or internet access, potentially introducing selection bias. However, we overcame this bias by conducting live sessions (face-to-face interviews) with around 50% of our included participants. Regarding the Cronbach’s alpha value for the intimate hygiene test, it was < 0.7, especially because the intimate hygiene questionnaire was a short one. The post-test assessments were administered within a relatively short time frame, specifically, within three hours following the awareness session. Consequently, we were unable to assess the long-term retention and application of the newly acquired information. Moreover, our research lacked an evaluation of potential changes in participants’ practices, particularly about screening, hygiene routines, and general lifestyle modifications. This aspect of follow-up could have been pivotal in understanding the sustained impact of our intervention and could have possibly served as a motivating factor for participants.

To mitigate the mentioned biases, our team was meticulously chosen and received thorough training to address the participants via face-to-face or phone interviews. Their role was to engage with participants effectively, enhance overall participation by offering necessary assistance in the online environment (50% of the population), and conducting live sessions to around 50% of the participants. On the other hand, the study’s *strengths* lie in its nationwide representative and multicentral sample, encompassing adult women participants of a wide age range from all Lebanese governorates and various backgrounds, providing a broad understanding of women’s health awareness. Being a longitudinal multicentric prospective interventional study with a diverse sample size fills a gap in the literature, contributing significantly to the community.

### 4.6. Impact and Perspectives

The findings emphasize the need for consistent attention to women’s health through educational initiatives. Breast cancer, cervical cancer, UTIs, and reproductive tract infections (RTIs)—significant public health concerns—are underrepresented and inadequately discussed, indicating a need for safe spaces to discuss sexual and reproductive health matters openly. Our study, encompassing essential and common topics of women’s health, is one of the first of its kind in Lebanon, especially because it included an interactive awareness session. Our results showed the positive impact this study had and could serve as a tool to encourage more engagement in this type of interventional studies, which not only assess the level of knowledge and practices, but work on improving them. We hypothesize that the provision of these awareness sessions throughout our study will enhance adherence to regular screenings and checkups and encourage the women attending these sessions to incorporate healthier habits into their daily routines. Future research should consider larger sample sizes and the inclusion of male participants. It is also valuable to assess the long-term assimilation of the newly acquired information by our participants, as well as the influence on their health practices, at 6 months and 1 year following the awareness sessions. Advocating for more national awareness campaigns is crucial to establish best practices and reduce the rates of breast and cervical cancer, optimizing reproductive health practices among women in Lebanon. This study could significantly influence the formulation and implementation of national health policies, serving as a basis for policy development aimed at addressing the knowledge gaps identified herein and ultimately promoting a healthier society.

## 5. Conclusions

This study questioned the impact of a community-based intervention on women’s awareness levels regarding breast cancer, cervical cancer, and intimate hygiene among women aged 18 to 83 in Lebanon. The study included 657 women across Lebanon’s 8 governorates, utilizing an analytical, longitudinal pre- and post-interventional design, resulting in a significant overall improvement of 50.48% in women’s health knowledge. The intervention emphasized screening, early treatment for breast and cervical cancer, reproductive health, and promoting healthy habits. Consequently, it is recommended to conduct awareness sessions in order to improve women’s knowledge and prevention measures and adherence to regular screenings, as well as to encourage them to adopt healthier habits. Furthermore, the financial constraints faced by the Lebanese economy in the last few years rendered access to knowledge and healthcare challenging for women, especially among vulnerable groups. Continuous follow-up on women’s assimilation of awareness session concepts and the adoption of healthy habits are essential for long-term impact. National awareness campaigns are crucial for establishing best practices reducing breast and cervical cancer rates and promoting healthier intimate hygiene, ultimately enhancing reproductive health among women in Lebanon. The study’s recommendations provide a framework for producing and updating evidence-based public health policies, namely, by the Ministry of Public Health and healthcare workers. Priority should be given to enhancing nationwide access to screening methods, considering their demonstrated effectiveness in early diagnosis and enhancement of women’s overall health. The recommendations provided above are intended to promote understanding of health-related topics and to dispel myths, taboos, and misconceptions surrounding breast cancer, cervical cancer, and intimate hygiene. Future research is needed to evaluate the level of adherence to regular screenings and its effect on reducing national breast cancer and cervical incidences as well as reducing intimate hygiene-related disease (UTIs, vaginitis, etc.).

## Figures and Tables

**Table 1:** Grading of knowledge and preventive measures in overall score, breast cancer, cervical cancer, and intimate hygiene, into categories of “Limited” and “Adequate” and subcategories of “Poor”, “Fair”, “Good”, and “Excellent”.

Categories	Subcategories	OVERALL SCORE		BREAST CANCER		CERVICAL CANCER		INTIMATE HYGIENE	
		Score/38	%	Score/18	%	Score/15	%	Score/5	%
LIMITED	POOR	≤23	≤60.52	≤11	≤61.11	≤9	≤60	≤3	≤60
	FAIR	[24–28]	[63.15–73.68]	[12–14]	[66.66–77.77]	[10–12]	[66.66–80]	[3–4]	[60–80]
ADEQUATE	GOOD	[29–35]	[76.31–92.1]	[15–17]	[83.33–94.44]	[13–14]	[86.66–93.33]	[4–5]	[80–100]
	EXCELLENT	≥36	≥94.7	18	100	15	100	-	-

**Table 2:** General characteristics of participants (in bold are represented the most prevalent parameters).

		Frequency	Percent
Nationality	Lebanese	**625**	**95.1**
	Others	32	4.9
Age	Mean (SD)	38.05 (14.07)	
	Min–Max	18–83	
Age	18–29 years	223	33.9
	30–44 years	216	32.9
	>44 years	218	33.2
Marital status	Single	239	36.4
	**Married**	**372**	**56.6**
	Divorced	16	2.4
	Widow	30	4.6
Governorate	Beirut	85	12.9
	Mount Lebanon	**228**	**34.7**
	Bekaa/Baalbek-Hermel	145	22.1
	North Lebanon/Akkar	105	16.0
	South Lebanon/Nabatiyeh	94	14.3
Highest level of education	I did not go to school	11	1.7
	Elementary school	81	12.3
	Bachelor’s degree	409	62.3
	Master’s degree	126	19.2
	MD/PhD	30	4.6
What type of work do you do?	Student	73	11.1
	Household labor	159	24.2
	Healthcare student	14	2.1
	Unemployed	93	14.2
	I work but not in the healthcare system	266	40.5
	I work in the healthcare system	52	7.9
Working status	No	339	51.6
	Yes	318	48.4

**Table 3:** Association between breast cancer K&P score and participants’ sociodemographic characteristics (age, governorate, Bekaa/Baalbek-Hermel governorate, highest level of education, type of work, working status, healthcare background, smoking, obesity) before and after attending the session (in bold are represented the *p*-value that are statistically significant *p* < 0.05).

BREAST CANCER	Variables	N	Before (Pre-Test)			After (Post-Test)		
			Mean	SD	*p*-Value	Mean	SD	*p*-Value
Nationality	Lebanese	625	10.60	3.45	0.472	16.13	2.25	0.871
	Others	32	10.16	2.90		16.06	1.87	
Age	18–29 years	223	10.78	3.28	0.105	16.38	1.75	0.093
	30–44 years	216	10.18	3.50		15.94	2.53	
	45–69 years	218	10.78	3.48		16.05	2.32	
Marital status	Single	239	11.09	3.12	**0.014**	16.49	1.77	**0.007**
	Married	372	10.29	3.60		15.87	2.46	
	Divorced	16	9.25	3.55		16.19	1.47	
	Widow	30	10.87	3.10		16.40	2.47	
Marital status	Not married	285	10.96	3.16	**0.013**	16.46	1.84	**<0.001**
	Married	372	10.29	3.60		15.87	2.46	
Governorate	Beirut	85	10.85	3.23	0.148	16.35	2.38	**0.016**
	Mount Lebanon	228	10.68	3.30		15.98	2.47	
	Bekaa/Baalbek-Hermel	145	10.17	3.57		16.61	0.99	
	North Lebanon/Akkar	105	11.11	3.64		15.84	2.95	
	South Lebanon/Nabatiyeh	94	10.15	3.38		15.84	1.74	
Governorate	Beirut/Mount Lebanon	313	10.72	3.28	0.316	16.08	2.45	0.622
	Others	344	10.45	3.56		16.17	2.01	
Highest level of education	I did not go to school	11	9.55	1.57	**<0.001**	15.00	3.26	**<0.001**
	Elementary school	81	9.84	3.36		14.81	3.48	
	Bachelor’s degree	409	10.37	3.44		16.18	2.01	
	Master’s degree	126	11.17	3.31		16.69	1.48	
	MD / PhD	30	13.30	2.93		16.90	1.35	
What type of work do you do?	Student	73	11.04	3.35	**<0.001**	16.49	1.74	**<0.001**
	Household labor	159	9.88	3.48		15.52	2.97	
	Healthcare student	14	11.50	3.01		16.79	1.37	
	Unemployed	93	9.97	4.36		15.65	2.12	
	I work but not in the healthcare system	266	10.59	3.00		16.45	1.89	
	I work in the healthcare system	52	12.87	2.56		16.50	1.60	
Working Status	No	339	10.22	3.72	**0.005**	15.81	2.50	**<0.001**
	Yes	318	10.97	3.04		16.46	1.84	
Do you smoke?	No	417	10.75	3.58	**0.041**	16.20	1.86	**<0.001**
	Ex-Smoker	135	10.67	3.30		16.57	2.32	
	Yes	105	9.81	2.82		15.26	3.07	
Obesity	No	560	10.57	3.35	0.783	16.26	2.11	**<0.001**
	Yes	97	10.67	3.87		15.36	2.69	
Presence of any comorbidity	No	403	10.67	3.33	0.379	16.37	1.93	**<0.001**
	Yes	254	10.43	3.58		15.74	2.60	
Did you previously receive any awareness campaign about breast cancer, cervical cancer or on women’s hygiene, in the last 6 months?	No	584	10.58	3.33	0.898	16.18	2.23	0.057
	Yes	73	10.63	4.15		15.66	2.17	

**Table 4:** Association between cervical cancer K&P score and participants’ sociodemographic characteristics (nationality, governorate, Bekaa/Baalbek-Hermel governorate, highest level of education, type of work, healthcare background) before and after attending the session (in bold are represented the *p*-value that are statistically significant *p* < 0.05).

CERVICAL CANCER	Variables	N	Before (Pre-Test)			After (Post-Test)		
			Mean	SD	*p*-Value	Mean	SD	*p*-Value
Nationality	Lebanese	625	7.96	2.92	**0.032**	12.84	1.54	0.324
	Others	32	9.09	2.91		12.56	1.16	
Age	18–29 years	223	8.01	2.91	0.926	12.78	1.67	0.602
	30–44 years	216	7.96	2.99		12.91	1.37	
	45–69 years	218	8.07	2.89		12.78	1.51	
Marital status	Single	239	8.08	2.74	0.882	12.96	1.64	0.322
	Married	372	7.94	3.05		12.73	1.47	
	Divorced	16	8.19	3.31		12.88	1.20	
	Widowed	30	8.30	2.68		12.90	1.32	
Marital status	Not married	285	8.11	2.75	0.483	12.95	1.59	0.065
	Married	372	7.94	3.05		12.73	1.47	
Governorate	Beirut	85	7.98	2.86	0.076	12.95	1.65	**0.009**
	Mount Lebanon	228	8.10	2.77		12.91	1.69	
	Bekaa/Baalbek-Hermel	145	8.28	3.25		13.05	0.90	
	North Lebanon/Akkar	105	8.19	2.82		12.49	1.80	
	South Lebanon/Nabatiyeh	94	7.23	2.88		12.52	1.31	
Governorate	Beirut/Mount Lebanon	313	8.07	2.79	0.656	12.92	1.68	0.115
	Others	344	7.97	3.05		12.73	1.36	
Highest level of education	I did not go to school	11	6.55	4.18	**<0.001**	11.82	2.96	**0.001**
	Elementary school	81	7.90	2.85		12.41	1.67	
	Bachelor’s degree	409	7.79	2.89		12.84	1.45	
	Master’s degree	126	8.26	2.82		12.94	1.49	
	MD/PhD	30	10.90	1.84		13.57	1.14	
What type of work do you do?	Student	73	7.81	2.81	**<0.001**	12.85	1.68	0.118
	Household labor	159	7.83	2.86		12.67	1.30	
	Healthcare student	14	8.71	2.55		12.43	1.65	
	Unemployed	93	7.05	3.53		12.57	1.82	
	I work but not in the healthcare system	266	8.16	2.71		12.98	1.49	
	I work in the healthcare system	52	9.65	2.60		13.00	1.41	
Working Status	No	339	7.65	3.05	**0.001**	12.67	1.55	**0.008**
	Yes	318	8.40	2.74		12.98	1.48	
Do you smoke?	No	417	8.01	3.08	0.615	12.80	1.49	**<0.001**
	Ex-Smoker	135	8.19	2.72		13.33	1.33	
	Yes	105	7.81	2.54		12.27	1.68	
Obesity	No	560	8.08	2.96	0.161	12.87	1.50	0.063
	Yes	97	7.63	2.73		12.56	1.65	
Presence of any comorbidity	No	403	8.10	2.91	0.346	12.90	1.43	0.085
	Yes	254	7.88	2.96		12.69	1.65	
Did you previously receive any awareness campaign about breast cancer, cervical cancer, or women’s hygiene in the last 6 months?	No	584	7.99	2.86	0.582	12.86	1.53	0.103
	Yes	73	8.19	3.46		12.55	1.48	

**Table 5:** Association between feminine hygiene K&P score and participants’ sociodemographic characteristics (highest level of education, type of work) and intimate hygiene score before and after attending the session (in bold are represented the *p*-value that are statistically significant *p* < 0.05).

FEMININE HYGIENE	Variables	N	Before (Pre-Test)			After (Post-Test)		
			Mean	SD	*p*-Value	Mean	SD	*p*-Value
Nationality	Lebanese	625	3.43	0.947	0.158	4.18	0.571	0.388
	Others	32	3.19	0.859		4.09	0.466	
Age	18–29 years	223	3.35	1.050	0.420	4.22	0.609	0.233
	30–44 years	216	3.46	0.919		4.19	0.605	
	45–69 years	218	3.44	0.847		4.13	0.472	
Marital status	Single	239	3.39	1.035	0.714	4.23	0.621	0.372
	Married	372	3.44	0.893		4.16	0.542	
	Divorced	16	3.19	1.047		4.06	0.574	
	Widowed	30	3.47	0.730		4.13	0.346	
Marital status	Not married	285	3.39	1.007	0.512	4.21	0.596	0.252
	Married	372	3.44	0.893		4.16	0.542	
Governorate	Beirut	85	3.48	0.895	0.683	4.12	0.730	0.351
	Mount Lebanon	228	3.46	0.959		4.19	0.551	
	Bekaa/Baalbek-Hermel	145	3.37	0.934		4.17	0.408	
	North Lebanon/Akkar	105	3.44	0.980		4.27	0.576	
	South Lebanon/Nabatiyeh	94	3.32	0.930		4.13	0.626	
Governorate	Beirut/Mount Lebanon	313	3.46	0.940	0.231	4.17	0.605	0.706
	Others	344	3.38	0.945		4.19	0.529	
Highest level of education	I did not go to school	11	3.45	0.820	**0.006**	4.18	0.405	0.111
	Elementary school	81	3.35	0.793		4.04	0.580	
	Bachelor’s degree	409	3.34	0.997		4.18	0.561	
	Master’s degree	126	3.61	0.848		4.23	0.568	
	MD/PhD	30	3.83	0.791		4.30	0.596	
What type of work do you do?	Student	73	3.27	1.158	0.176	4.26	0.553	0.170
	Household labor	159	3.35	0.857		4.10	0.553	
	Healthcare student	14	3.07	1.141		4.14	0.864	
	Unemployed	93	3.48	0.928		4.13	0.423	
	I work but not in the healthcare system	266	3.45	0.936		4.23	0.592	
	I work in the healthcare system	52	3.62	0.844		4.13	0.595	
Working Status	No	339	3.36	0.960	0.092	4.14	0.538	0.117
	Yes	318	3.48	0.922		4.21	0.593	
Do you smoke?	No	417	3.46	0.955	0.156	4.22	0.582	**0.032**
	Ex-Smoker	135	3.42	0.868		4.07	0.398	
	Yes	105	3.26	0.981		4.15	0.662	
Obesity	No	560	3.40	0.961	0.319	4.20	0.562	**0.046**
	Yes	97	3.51	0.831		4.07	0.582	
Presence of any comorbidity	No	403	3.43	0.978	0.739	4.22	0.591	**0.031**
	Yes	254	3.40	0.887		4.12	0.520	
Did you previously receive any awareness campaign about breast cancer, cervical cancer, or women’s hygiene in the last 6 months?	No	584	3.42	0.936	0.748	4.19	0.564	0.188
	Yes	73	3.38	1.009		4.10	0.581	

**Table 6:** Association between K&P overall score and participants’ sociodemographic characteristics (age; governorate; Bekaa/Baalbek-Hermel governorate; highest level of education; type of work; working status; healthcare background; smoking; obesity; previous reception of an awareness session concerning breast cancer, cervical cancer, or intimate hygiene) before and after attending the session (in bold are represented the *p*-value that are statistically significant *p* < 0.05).

OVERALL	Variables	N	Before (Pre-Test)			After (Post-Test)		
			Mean	SD	*p*-Value	Mean	SD	*p*-Value
Nationality	Lebanese	625	21.99	5.982	0.679	33.15	3.431	0.490
	Others	32	22.44	5.273		32.72	2.954	
Age	18–29 years	223	22.15	5.917	0.436	33.38	3.204	0.391
	30–44 years	216	21.59	6.213		33.03	3.583	
	45–69 years	218	22.29	5.707		32.96	3.435	
Marital status	Single	239	22.56	5.608	0.220	33.67	3.207	**0.011**
	Married	372	21.67	6.153		32.75	3.508	
	Divorced	16	20.63	6.771		33.13	2.680	
	Widowed	30	22.63	5.321		33.43	3.569	
Marital status	Not married	285	22.46	5.645	0.094	33.62	3.213	**0.001**
	Married	372	21.67	6.153		32.75	3.508	
Governorate	Beirut	85	22.31	5.559	0.145	33.42	3.778	**0.012**
	Mount Lebanon	228	22.23	5.650		33.07	3.815	
	Bekaa/Baalbek-Hermel	145	21.81	6.491		33.83	1.717	
	North Lebanon/ Akkar	105	22.74	6.100		32.59	4.206	
	South Lebanon/Nabatiyeh	94	20.70	5.844		32.49	2.754	
Governorate	Beirut/Mount Lebanon	313	22.25	5.617	0.324	33.17	3.802	0.750
	Others	344	21.79	6.232		33.08	3.010	
Highest level of education	I did not go to school	11	19.55	4.906	**<0.001**	31.00	6.197	**<0.001**
	Elementary school	81	21.09	5.446		31.26	4.690	
	Bachelor’s degree	409	21.50	5.914		33.21	3.060	
	Master’s degree	126	23.05	5.904		33.86	2.836	
	MD/PhD	30	28.03	4.123		34.77	2.402	
What type of work do you do?	Student	73	22.12	6.148	**<0.001**	33.60	2.876	**<0.001**
	Household labor	159	21.06	5.775		32.28	3.704	
	Healthcare student	14	23.29	5.770		33.36	3.342	
	Unemployed	93	20.51	7.671		32.34	3.631	
	I work but not in the healthcare system	266	22.21	5.115		33.66	3.246	
	I work in the healthcare system	52	26.13	4.757		33.63	2.904	
Working Status	No	339	21.23	6.438	**<0.001**	32.63	3.536	**<0.001**
	Yes	318	22.85	5.256		33.65	3.188	
Do you smoke?	No	417	22.21	6.320	0.101	33.21	3.056	**<0.001**
	Ex-Smoker	135	22.28	5.639		33.98	3.320	
	Yes	105	20.88	4.559		31.68	4.316	
Obesity	No	560	22.05	5.920	0.709	33.32	3.249	**<0.001**
	Yes	97	21.80	6.125		31.99	4.045	
Presence of any comorbidity	No	403	22.20	5.915	0.306	33.49	2.974	**0.001**
	Yes	254	21.71	5.997		32.55	3.941	
Did you previously receive any awareness campaign about breast cancer, cervical cancer, or women’s hygiene in the last 6 months?	No	584	21.99	5.720	0.769	33.23	3.383	**0.028**
	Yes	73	22.21	7.566		32.30	3.519	

## Data Availability

The data presented in this study are available on request from the corresponding author and OML. The data are not publicly available due to their containing information that could compromise the privacy of research participants.

## Supplementary Materials

The following supporting information can be downloaded at: https://www.mdpi.com/article/10.3390/healthcare12232422/s1, Figure S1. Schematic representation of the mean percentage (%) of women’s answers before and after the awareness campaign about the three topics overall, breast cancer, cervical cancer, and intimate (feminine) hygiene, organized under subcategories “Poor/Fair/Good/Excellent”. Each rectangle represents 100% of the answers. Table S1. Table summarizing pre- and post-tests for all K&P scores (overall, breast cancer, cervical cancer, and intimate (feminine) hygiene) with mean, median, and standard deviation. Table S2. Percentages (%) of participants’ improvement and correct answers to pre- and post-test about breast cancer. Table S3. Percentages (%) of participants’ improvement and correct answers to pre- and post-test about cervical cancer. Table S4. Percentages (%) of participants’ improvement and correct answers to pre- and post-test about intimate (feminine) hygiene. Table S5. Multivariate analysis of the overall K&P score before and after the awareness session about breast cancer, cervical cancer, and intimate hygiene.
