# Supplementary material for: The Impact of a Randomized Community-Based Intervention on the Awareness of Women Residing in Lebanon Toward Breast Cancer, Cervical Cancer, and Intimate Hygiene
Source: Healthcare (Basel). 2024 Dec 3;12(23):2422. doi: 10.3390/healthcare12232422 (PMC11641698; doi:10.3390/healthcare12232422)
Supplement: Supplementary file 1 [file healthcare-12-02422-s001.zip › SUPPLEMENTARY MATERIALS + FIG S1/Supplementary Materials- Questionnaire.docx]

**QUESTIONNAIRE**

**‘’The Impact Of A Randomized Community-Based Intervention On The Awareness Of Women Residing In Lebanon Toward Breast Cancer, Cervical Cancer, And Intimate Hygiene”**

Please type in here the "Identification Number (ID)" **code** that has been assigned to you

__________

# **CONSENT**

Do you agree to participate:

- Yes
- No

# **SOCIO DEMOGRAPHIC CHARACTERISTICS**

1. Nationality:
2. Lebanese
3. Syrian
4. Other: __________
5. Year of birth:

__________

1. How old are you?

__________

1. Please select your marital status:
2. Single
3. Married
4. Divorced
5. Widow
6. Please specify the governorate you live in:
7. Beirut
8. Mount Lebanon
9. North Lebanon
10. South Lebanon
11. Bekaa
12. Akkar
13. Nabatiyeh
14. Baalbek-Hermel
15. Please select the highest level of education you have completed. And in case you are still completing your studies, choose the degree you are currently enrolled in (the one you are currently studying)
16. I did not go to school
17. Elementary school
18. Baccalaureate
19. Bachelor’s degree (or 2^nd^ and 3^rd^ year medical studies)
20. Master’s degree (or 4^th^ and 5^th^ year medical studies)
21. PhD
22. Medical degree
23. What type of work do you do?
24. I work in the healthcare system
25. I work but not in the healthcare system
26. Unemployed
27. Household labor
28. Student
29. Healthcare student
30. What chronic diseases do (or did) you suffer from?

|  | Yes | No |
| --- | --- | --- |
| Diabetes mellitus |  |  |
| Cardiovascular disease |  |  |
| Hypertension |  |  |
| High cholesterol |  |  |
| Obesity |  |  |
| Thyroid disease |  |  |
| Stroke |  |  |
| Coronary artery disease |  |  |
| Lung diseases (COPD, asthma…) |  |  |
| Renal failure |  |  |
| Breast cancer |  |  |
| Cervical cancer |  |  |
| Other types of cancers |  |  |
| Other |  |  |

1. Do you smoke?
2. Yes
3. No
4. Ex-smoker for less than 15 years
5. Ex-smoker for more than 15 years
6. What is you weight? In kilograms, Kg

__________

1. What is your height? In centimeters, cm

__________

1. Did you previously receive any awareness campaign about breast cancer, cervical cancer or on women’s hygiene, in the last 6 months?
2. Yes
3. No

# **PRE-TEST KNOWLDEGE & PRACTICE ABOUT BREAST CANCER**

Please do not get assistance neither from the internet nor from a book. Please just respond according to "your" knowledge!

1. Identify which of the following is a warning sign of breast cancer

|  | Yes | No | Don’t know |
| --- | --- | --- | --- |
| Lump or thickening under the armpit |  |  |  |
| Bleeding or discharge from the nipple |  |  |  |
| Rash on or around the nipple |  |  |  |
| Redness of the breast skin |  |  |  |
| Change in the size of the breast or nipple |  |  |  |
| Change in the shape of the breast or nipple |  |  |  |

1. The percentage of healing and recovery from breast cancer is more than 90%
2. True
3. False
4. Identify the risk factors of breast cancer

|  | Yes | No | Don’t know |
| --- | --- | --- | --- |
| Family history of breast or ovarian cancer |  |  |  |
| Early menstruation |  |  |  |
| Breastfeeding |  |  |  |
| Never being pregnant |  |  |  |
| Alcohol consumption |  |  |  |
| Young age |  |  |  |
| Obesity |  |  |  |
| Hormone therapy |  |  |  |
| Low physical activity |  |  |  |

1. No need to consult your doctor in case you find an abnormal mass in your breast that is not painful
2. True
3. False
4. In females with high risk of breast cancer, annual screening using MRI and mammogram should begin at the age of 40
5. True
6. False

# **PRE-TEST KNOWLEDGE & PRACTICE ABOUT CERVICAL CANCER**

Please do not get assistance neither from the internet nor from a book. Please just respond according to "your" knowledge!

1. Does a pap smear detect cervical cancer?
2. Yes
3. No
4. Don’t know
5. Human Papillomavirus (HPV) plays a role in the etiology of cervical cancer
6. Yes
7. No
8. Don’t know
9. HPV is sexually transmitted, especially by close skin contact
10. Yes
11. No
12. Don’t know
13. What are the main risk factors for cervical cancer?

|  | Yes | No | Don’t know |
| --- | --- | --- | --- |
| Weak immune system |  |  |  |
| Smoking |  |  |  |
| Having kids at an early age |  |  |  |
| Having one of the ovaries removed |  |  |  |
| Stress |  |  |  |

1. Identify the symptoms of cervical cancer

|  | Yes | No | Don’t know |
| --- | --- | --- | --- |
| Constant back pain |  |  |  |
| Tingling in the foot |  |  |  |
| Postmenopausal spotting or bleeding |  |  |  |
| Heavy bleeding between periods in females of childbearing age |  |  |  |
| Bleeding after sexual intercourse |  |  |  |

1. “If I get HPV vaccine, I also need regular screening for cervical cancer”
2. True
3. False
4. How often should a woman with low-risk of cervical cancer perform a pap smear?
5. Once every 6 months
6. Once per year
7. Once every 3 years

# **PRE-TEST KNOWLEDGE & PRACTICE ABOUT WOMEN’S HYGIENE**

Please do not get assistance neither from the internet nor from a book. Please just respond according to "your" knowledge!

1. Douching is necessary as it keeps the natural balance of healthy bacteria living inside the vagina
2. True
3. False
4. Use of antibiotics or exposure to a lot of stress can induce overgrowth of fungi that are naturally present in the body, thus resulting in reproductive tract infections
5. True
6. False
7. To prevent vaginal infections, do you wear:
8. Cotton underwear
9. Silk underwear
10. It does not matter
11. What do you use when washing?
12. The hand with water and a specific hygiene product
13. The loofah
14. Washcloth (Gant de toilette)
15. After urinating and after defecation, you should wipe:
16. From back to front
17. From front to back
18. It does not matter: as far as it is well wiped

# **POST-TEST KNOWLEDGE & PRACTICE ABOUT BREAST CANCER**

Please do not get assistance neither from the internet nor from a book. Please just respond according to "your" knowledge!

1. Identify which of the following is a warning sign of breast cancer

|  | Yes | No | Don’t know |
| --- | --- | --- | --- |
| Lump or thickening under the armpit |  |  |  |
| Bleeding or discharge from the nipple |  |  |  |
| Rash on or around the nipple |  |  |  |
| Redness of the breast skin |  |  |  |
| Change in the size of the breast or nipple |  |  |  |
| Change in the shape of the breast or nipple |  |  |  |

1. The percentage of healing and recovery from breast cancer is more than 90%
2. True
3. False
4. Identify the risk factors of breast cancer

|  | Yes | No | Don’t know |
| --- | --- | --- | --- |
| Family history of breast or ovarian cancer |  |  |  |
| Early menstruation |  |  |  |
| Breastfeeding |  |  |  |
| Never being pregnant |  |  |  |
| Alcohol consumption |  |  |  |
| Young age |  |  |  |
| Obesity |  |  |  |
| Hormone therapy |  |  |  |
| Low physical activity |  |  |  |

1. No need to consult your doctor in case you find an abnormal mass in your breast that is not painful
2. True
3. False
4. In females with high risk of breast cancer, annual screening using MRI and mammogram should begin at the age of 40
5. True
6. False

# **POST-TEST KNOWLEDGE & PRATCICE ABOUT CERVICAL CANCER**

Please do not get assistance neither from the internet nor from a book. Please just respond according to "your" knowledge!

1. Does a pap smear detect cervical cancer?
2. Yes
3. No
4. Don’t know
5. Human Papillomavirus (HPV) plays a role in the etiology of cervical cancer
6. Yes
7. No
8. Don’t know
9. HPV is sexually transmitted, especially by close skin contact
10. Yes
11. No
12. Don’t know
13. What are the main risk factors for cervical cancer?

|  | Yes | No | Don’t know |
| --- | --- | --- | --- |
| Weak immune system |  |  |  |
| Smoking |  |  |  |
| Having kids at an early age |  |  |  |
| Having one of the ovaries removed |  |  |  |
| Stress |  |  |  |

1. Identify the symptoms of cervical cancer

|  | Yes | No | Don’t know |
| --- | --- | --- | --- |
| Constant back pain |  |  |  |
| Tingling in the foot |  |  |  |
| Postmenopausal spotting or bleeding |  |  |  |
| Heavy bleeding between periods in females of childbearing age |  |  |  |
| Bleeding after sexual intercourse |  |  |  |

1. “If I get HPV vaccine, I also need regular screening for cervical cancer”
2. True
3. False
4. How often should a woman with low-risk of cervical cancer perform a pap smear?
5. Once every 6 months
6. Once per year
7. Once every 3 years

# **POST-TEST KNOWLEDGE & PRACTICE ABOUT FEMININE HYGIENE**

Please do not get assistance neither from the internet nor from a book. Please just respond according to "your" knowledge!

1. Douching is necessary as it keeps the natural balance of healthy bacteria living inside the vagina
2. True
3. False
4. Use of antibiotics or exposure to a lot of stress can induce overgrowth of fungi that are naturally present in the body, thus resulting in reproductive tract infections
5. True
6. False
7. To prevent vaginal infections, do you wear:
8. Cotton underwear
9. Silk underwear
10. It does not matter
11. What do you use when washing?
12. The hand with water and a specific hygiene product
13. The loofah
14. Washcloth (Gant de toilette)
15. After urinating and after defecation, you should wipe:
16. From back to front
17. From front to back
18. It does not matter: as far as it is well wiped

***Thank you for your participation!***
