# Supplementary material for: The Impact of a Randomized Community-Based Intervention on the Awareness of Women Residing in Lebanon Toward Breast Cancer, Cervical Cancer, and Intimate Hygiene
Source: Healthcare (Basel). 2024 Dec 3;12(23):2422. doi: 10.3390/healthcare12232422 (PMC11641698; doi:10.3390/healthcare12232422)
Supplement: Supplementary file 1 [file healthcare-12-02422-s001.zip › SUPPLEMENTARY MATERIALS + FIG S1/Supplementary TABLES S1 S 2 S3 S4 S5.docx]

# **Supplementary tables**

Table S1. Table summarizing pre and post-tests for all K& P scores (overall, breast cancer, cervical cancer, and feminine hygiene) with mean, median and std deviation.

|  | | Pre Overall Score | Pre Breast Cancer test | Pre Cervical Cancer test | Pre Woman Hygiene test | Post Overall Score | Post Breast Cancer test | Post Cervical Cancer test | Post Woman Hygiene test |
| --- | --- | --- | --- | --- | --- | --- | --- | --- | --- |
| N | Valid | 657 | 657 | 657 | 657 | 657 | 657 | 657 | 657 |
|  | Missing | 0 | 0 | 0 | 0 | 0 | 0 | 0 | 0 |
| Mean | | 22.01 | 10.58 | 8.01 | 3.42 | 33.12 | 16.12 | 12.82 | 4.18 |
| Median | | 23.00 | 11.00 | 8.00 | 4.00 | 34.00 | 17.00 | 13.00 | 4.00 |
| Std. Deviation | | 5.95 | 3.43 | 2.93 | 0.94 | 3.41 | 2.23 | 1.52 | 0.57 |
| Minimum | | 1 | 0 | 0 | 0 | 15 | 2 | 5 | 2 |
| Maximum | | 35 | 18 | 15 | 5 | 38 | 18 | 15 | 5 |
| Percentiles | 25 | 19.00 | 9.00 | 6.00 | 3.00 | 32.00 | 16.00 | 12.00 | 4.00 |
|  | 50 | 23.00 | 11.00 | 8.00 | 4.00 | 34.00 | 17.00 | 13.00 | 4.00 |
|  | 75 | 26.00 | 13.00 | 10.00 | 4.00 | 35.00 | 17.00 | 14.00 | 5.00 |
| **Score over** | | 38 | 18 | 15 | 5 | 38 | 18 | 15 | 5 |

Table S2. Percentages (%) of participants’ improvement and correct answers to pre- and post-test about Breast Cancer.

| **Breast Cancer** | | | | |
| --- | --- | --- | --- | --- |
| **Score** | | | | |
| **Warning signs of Breast cancer identification** | | | | |
|  |  | Pre-test | Post-test | Improvement |
| Lump or thickening under armpit | | 74.3% | 92.2% | 24.2% |
| Bleeding or discharge from the nipple | | 72.1% | 96.5% | 33.8% |
| Rash on or around the nipple | | 51.4% | 90.6% | 76.0% |
| Redness of the breast skin | | 52.5% | 90.9% | 73.0% |
| Change in the size of the breast or nipple | | 65.9% | 97.0% | 47.1% |
| Change in the shape of the breast or nipple | | 67.1% | 96.8% | 44.2% |
| **Identification of Breast cancer risk factor** | | | | |
|  |  | Pre-test | Post-test | Improvement |
| Family history of breast or ovarian cancer | | 88.6% | 98.2% | 10.8% |
| Early menstruation | | 16.1% | 84.2% | 421.7% |
| Breastfeeding | | 81.4% | 88.3% | 8.4% |
| Never being pregnant | | 24.2% | 85.5% | 253.5% |
| Alcohol consumption | | 50.1% | 92.7% | 85.1% |
| Young age | | 77.2% | 77.9% | 1.00% |
| Obesity | | 40.9% | 91.6% | 123.8% |
| Hormone therapy | | 60.0% | 95.1% | 58.7% |
| Low physical activity | | 52.8% | 92.2% | 74.7% |
| **Answering correctly with “FALSE” concerning Breast Cancer misinformation** | | | | |
|  |  | Pre-test | Post-test | Improvement |
| “In females with high risk breast cancer, annual screening using MRI and mammogram should begin at the age of 40” | | 30.1% | 55.7% | 84.8% |
| “No need to consult your doctor in case you find an abnormal mass in your breast that is not painful” | | 87.7% | 90.0% | 2.1% |
| **“The percentage of healing and recovery from breast cancer is more than 90%”** | | | | |
|  |  | Pre-test | Post-test | Improvement |
| True | | 65.6% | 97.1% | 48.0% |
| False | | 34.4% | 2.9% | -91.6% |

Table S3. Percentages (%) of participants’ improvement and correct answers to pre- and post-test about Cervical Cancer.

| **Cervical cancer** | | | | |
| --- | --- | --- | --- | --- |
| **Identification of symptoms of cervical cancer** | | | | |
|  |  | Pre-test | Post-test | Improvement |
| Bleeding after sexual intercourse | | 58.0% | 96.0% | 62.0% |
| Heavy bleeding between periods in females of childbearing age | | 71.2% | 91.2% | 35.3% |
| Postmenopausal spotting or bleeding | | 82.0% | 98.9% | 20.6% |
| Tingling in foot | | 22.1% | 96.3% | 313.1% |
| Constant back pain | | 44.0% | 93.9% | 118.4% |
| **Main risk factors of cervical cancer** | | | | |
|  |  | Pre-test | Post-test | Improvement |
| Weak immune system | | 68.3% | 89.0% | 30.3% |
| Smoking | | 58.1% | 96.2% | 65.4% |
| Having one of the ovaries removed | | 31.5% | 46.6% | 47.8% |
| Having kids at an early age | | 49.0% | 47.6% | -2.80% |
| Stress | | 61.6% | 90.3% | 46.4% |
| **“How often should a woman with low-risk of cervical cancer perform a pap smear?”** | | | | |
|  |  | Pre-test | Post-test | Improvement |
| Once every 6 months | | 21.3% | 4.4% | -79.3% |
| Once per year | | 62.3% | 43.8% | -29.58% |
| Once every 3 years | | 16.4% | 50.5% | 207.4% |
| **“If I get HPV vaccine, I also need regular screening for cervical cancer”** | | | | |
|  |  | Pre-test | Post-test | Improvement |
| True | | 81.0% | 96.3 | 19.0% |
| False | | 19.0% | 3.7% | -80.8% |
| **“HPV is one of the main reason of developing Cervical Cancer”** | | | | |
|  |  | Pre-test | Post-test | Improvement |
| Yes | | 43.2% | 95.9% | 121.9% |
| No | | 2.7% | 1.8% | -33.4% |
| I don’t know | | 54.0% | 2.3% | -95.8% |
| **Knowledge about pap smear detecting cervical cancer** | | | | |
|  |  | Pre-test | Post-test | Improvement |
| Yes | | 70.0% | 98.6% | 40.9% |
| No | | 4.1% | 0.8% | -81.5% |
| I don’t know | | 25.9% | 0.6% | -97.7% |
| **“HPV is sexually transmitted especially by close skin contact”** | | | | |
|  |  | Pre-test | Post-test | Improvement |
| Yes | | 44.7% | 94.7% | 111.6% |
| No | | 11.1% | 3.0% | -72.6% |
| I don’t know | | 44.1% | 2.3% | -94.8% |

Table S4. Percentages (%) of participants’ improvement and correct answers to pre- and post-test about Feminine hygiene.

| **Feminine Hygiene** | | | | |
| --- | --- | --- | --- | --- |
| **“What do you wear to prevent vaginal infection?”** | | | | |
|  |  | Pre-test | Post-test | Improvement |
| Cotton underwear | | 86.5% | 96.8% | 12.0% |
| Silk underwear | | 4.0% | 1.1% | -73.1% |
| It does not matter | | 9.6% | 2.1% | -77.8% |
| **“What do you use when washing?”** | | | | |
|  |  | Pre-test | Post-test | Improvement |
| The hand with water and a specific hygiene product | | 73.7% | 97.3% | 32.0% |
| The loofah | | 11.0% | 1.1% | -90.3% |
| Washcloth (gant de toilette) | | 15.4% | 1.7% | -89.1% |
| **“After urinating and after defecation, how should you wipe?”** | | | | |
|  |  | Pre-test | Post test | Improvement |
| From back to front | | 7.3% | 2.3% | -68.7% |
| From front to back | | 93.1% | 97.3% | 17.0% |
| it does not matter as far as it is well wiped | | 9.6% | 0.5% | -95.2% |
| **“Use of antibiotics or exposure to a lot of stress can induce overgrowth of fungi that are naturally present in the body, thus resulting in reproductive tract infections”** | | | | |
|  |  | Pre-test | Post-test | Improvement |
| True | | 83.1% | 97.0% | 16.7% |
| False | | 16.9% | 3.0% | -82.0% |
| **“Douching is necessary as it keeps the natural balance of living bacteria living inside the vagina”** | | | | |
|  |  | Pre-test | Post-test | Improvement |
| True | | 84.6% | 70.5% | -16.7% |
| False | | 15.4% | 29.5% | 92.1% |

**Table S5.** Multivariate analysis of the overall K&P score before and after the awareness session about breast cancer, cervical cancer and intimate hygiene

| **Pre-test** | Model | | Unstandardized Coefficients | | Standardized Coefficients | t | Sig. | 95.0% Confidence Interval for B | | Collinearity Statistics | |
| --- | --- | --- | --- | --- | --- | --- | --- | --- | --- | --- | --- |
|  |  |  | B | Std. Error | Beta |  |  | Lower Bound | Upper Bound | Tolerance | VIF |
|  | 2 | **(Constant)** | 16.664 | 0.981 |  | 16.994 | 0.000 | 14.738 | 18.589 |  |  |
|  |  | **Highest level of education** | 1.547 | 0.315 | 0.193 | 4.914 | **0.000** | 0.929 | 2.165 | 0.939 | 1.064 |
|  |  | **Working Status** | 1.058 | 0.467 | 0.089 | 2.267 | **0.024** | 0.142 | 1.974 | 0.939 | 1.064 |
|  | a.      Dependent Variable: Pre-test score | | | | | | | | | | |
|  |  | | | | | | | | | | |
| **Post-test** | Model | | Unstandardized Coefficients | | Standardized Coefficients | t | Sig. | 95.0% Confidence Interval for B | | Collinearity Statistics | |
|  |  |  | B | Std. Error | Beta |  |  | Lower Bound | Upper Bound | Tolerance | VIF |
|  | 5 | **(Constant)** | 30.532 | 0.602 |  | 50.694 | 0.000 | 29.349 | 31.714 |  |  |
|  |  | **Highest level of education** | 0.918 | 0.180 | 0.200 | 5.097 | **0.000** | 0.564 | 1.271 | 0.911 | 1.097 |
|  |  | **Obesity** | -1.001 | 0.362 | -0.104 | -2.763 | **0.006** | -1.713 | -0.290 | 0.981 | 1.020 |
|  |  | **Any awareness campaign on 3 topics in the last 6 months?** | -0.906 | 0.409 | -0.084 | -2.215 | **0.027** | -1.709 | -0.103 | 0.981 | 1.020 |
|  |  | **Working Status** | 0.555 | 0.264 | 0.081 | 2.098 | **0.036** | 0.036 | 1.074 | 0.928 | 1.078 |
|  |  | **Marital status** | -0.521 | 0.262 | -0.076 | -1.994 | **0.047** | -1.035 | -0.008 | 0.965 | 1.036 |
