# Supplementary figures and images for: The Impact of a Randomized Community-Based Intervention on the Awareness of Women Residing in Lebanon Toward Breast Cancer, Cervical Cancer, and Intimate Hygiene
Source: Healthcare (Basel). 2024 Dec 3;12(23):2422. doi: 10.3390/healthcare12232422 (PMC11641698; doi:10.3390/healthcare12232422)

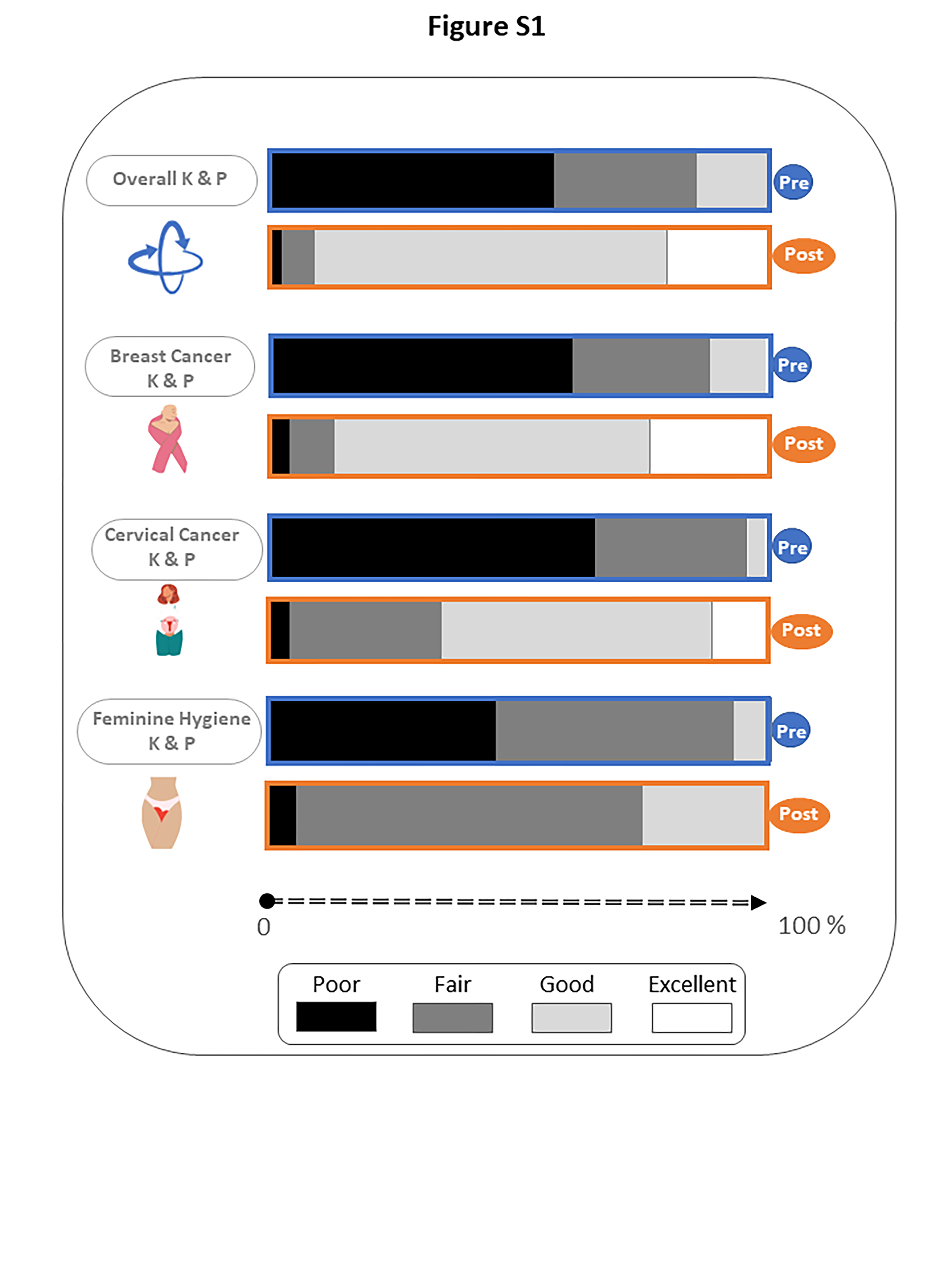

Supplement: Supplementary file 1 [file healthcare-12-02422-s001.zip › SUPPLEMENTARY MATERIALS + FIG S1/Figure S1 TIFF.tif]
